# Supplementary material for: Controlling congestion on complex networks: fairness, efficiency and network structure
Source: Sci Rep. 2017 Aug 22;7:9152. doi: 10.1038/s41598-017-09524-3 (PMC5567293; doi:10.1038/s41598-017-09524-3)
Supplement: Supplementary file 1 — Supplementary Information [file 41598_2017_9524_MOESM1_ESM.pdf]

Supplementary Information: Controlling congestion on complex networks: fairness, efficiency and network structure

Lubos Buzna and Rui Carvalho

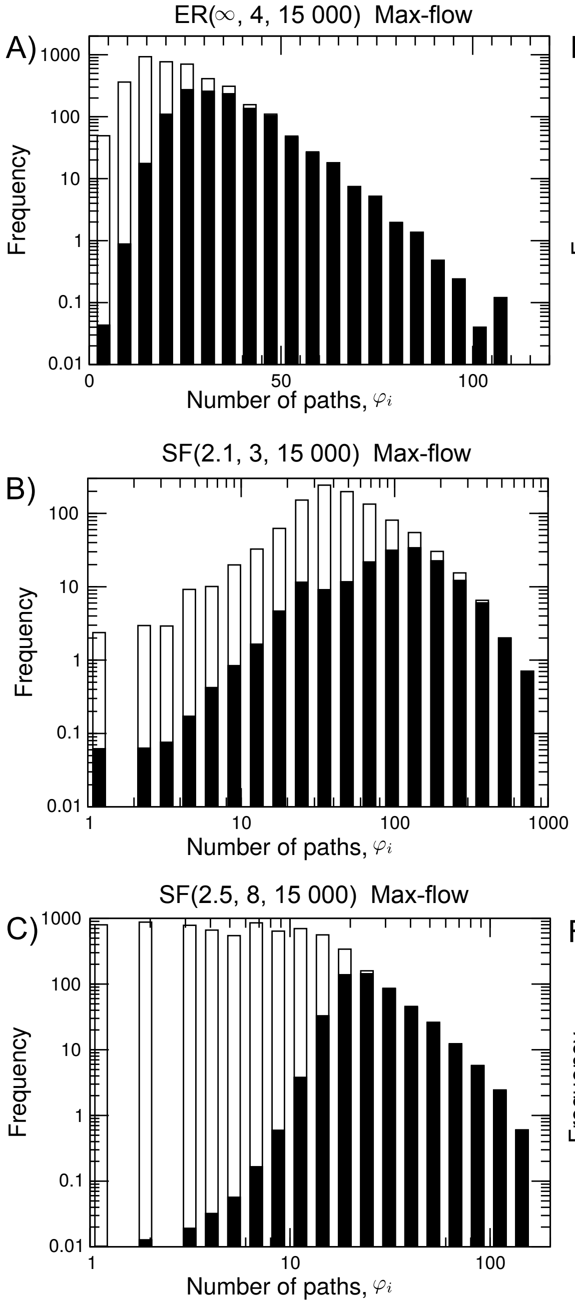

Figure S1. (A-C) Histograms of $\varphi(N, i)$ values computed from 20 random realisations and $N = 2000$ for ER(∞, 4, 15 000) networks and two classes of SF networks corresponding to the two highlighted cells in Fig. 3B-D, i.e., SF(2.1, 3, 15 000) and SF(2.5, 8, 15 000). Panels A-C (D-E) show histograms for the max-flow (max-min fairness), where the shaded area of each bin is the proportion of bottleneck edges in the bin. Bottlenecks are saturated edges, that is edges for which $F_i \geq 0.9999c$, where c is edge capacity.
